# Metastatic Plasmacytoid Urothelial Carcinoma Masquerading as a Primary Signet Ring Cell Carcinoma of the Duodenum

**DOI:** 10.7759/cureus.44454

**Published:** 2023-08-31

**Authors:** Adil S Mir, Mohamad Mouchli, David P Lebel, Douglas Grider

**Affiliations:** 1 Gastroenterology, Virginia Tech Carilion School of Medicine, Roanoke, USA; 2 Gastroenterology and Hepatology, Cleveland Clinic Foundation, Cleveland, USA; 3 Pathology, Dominion Pathology Associates, Roanoke, USA; 4 Basic Science Education, Virginia Tech Carilion School of Medicine, Roanoke, USA; 5 Pathology, Carilion Roanoke Memorial Hospital, Roanoke, USA

**Keywords:** small bowel malignancy, rare cancers, cd-138, pitfall, urothelial malignancy, plasmacytoid

## Abstract

Plasmacytoid urothelial carcinoma can histologically mimic gastrointestinal signet ring cell carcinoma, a potential diagnostic pitfall resulting in improper clinical management. We present a rare case of a malignant duodenal ulcer due to metastasis from plasmacytoid urothelial carcinoma. Only by histological and retrospective immunohistochemical comparison with the primary bladder tumor was this revealed as a metastasis from a plasmacytoid urothelial carcinoma. This case report highlights the importance of clinical correlation and comparison with any previous pathology specimens, the limitations of immunohistochemical staining, and the utilization of both old and new immunohistochemical tools when differentiating signet ring cell carcinomas of primary sites versus potential metastases.

## Introduction

Urothelial carcinoma is common in developed countries and is related to smoking and carcinogen exposures. The plasmacytoid variant of urothelial carcinoma has an aggressive clinical course, usually presenting late with nontraditional metastases along peritoneal surfaces and fascial planes, and is more prone to recurrence. This variant can mimic the gastrointestinal (GI) signet ring carcinoma, and thus, metastasis to the tubular GI tract can be easily missed. Presented herein is a duodenal metastasis from a plasmacytoid urothelial carcinoma (PUC), which at initial evaluation was considered to be a primary small bowel signet ring cell carcinoma because of the immunoprofile, which included negative GATA binding protein 3 (GATA3) and cytokeratin (CK) 20, two immunohistochemical stains usually positive in urothelial carcinomas. GATA3 is usually positive in breast carcinoma as well and is a more likely metastasis to the small intestine than high-grade urothelial carcinoma. However, the prior transurethral resection of the prostate (TURP) specimen was retrieved and compared to the duodenal specimen to make the diagnosis, showing the importance of clinical history and comparison with prior surgical pathology specimens.

## Case presentation

**Figure 1:**
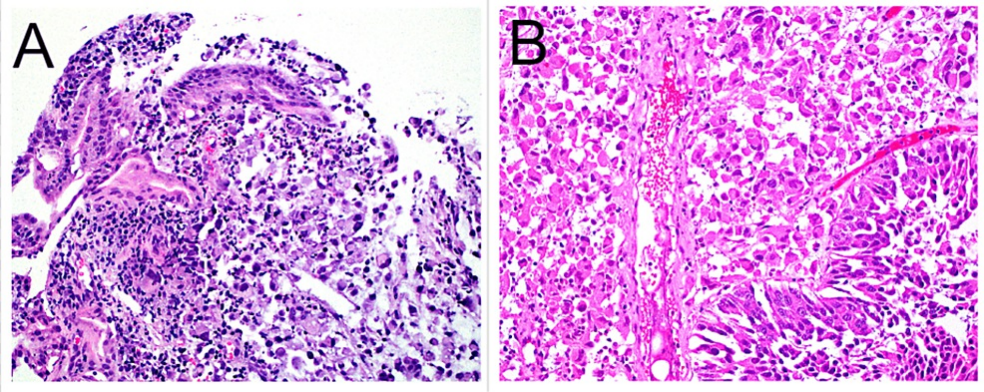
(A) Duodenal biopsy showing nondysplastic small bowel surface epithelium with distorted villous architecture (left) and lamina propria expanded by malignant cells with dyscohesive growth, eccentric and hyperchromatic nuclei, eosinophilic cytoplasm, and signet ring-like cell forms, H&E at 20x (200 magnification). (B) Transurethral resection specimen showing invasive urothelial carcinoma with both conventional phenotype growth (bottom right) and plasmacytoid variant histology (upper left) identical to the malignant cells seen in the duodenal biopsy (A), H&E at 20x (200 magnification). H&E: hematoxylin and eosin.

A 66-year-old female with a past medical history of hypertension, diabetes mellitus, and hyperlipidemia presented to the emergency department (ED) with weakness and anemia (hemoglobin 5.2 g/dL; normal range 12-16 g/dL). Computed tomography (CT) demonstrated distal duodenal wall thickening, a large 8.2 x 5.7 x 4.3 cm right adrenal mass, and pelvic lymphadenopathy. Esophagogastroduodenoscopy (EGD) revealed a duodenal ulcer with rolled borders and central ulceration with visible vessels. Biopsies demonstrated nondysplastic small bowel mucosa with a dyscohesive cellular infiltrate expanding the lamina propria, resembling signet ring cells (Figure 1A). An initial panel of immunohistochemical stains was performed to characterize this infiltrate. There was positivity for CK7, but negativity for CK20 and CDX-2, an immunoprofile not incompatible with either a potential small bowel primary or metastatic signet ring cell carcinoma.

A chart review suggested that about a year ago the patient had been found to have a 6.0 x 4.3 x 4.8 cm irregular solid mass along the posterior bladder wall on ultrasound, and subsequent CT imaging showed this same bladder mass to have transmural extension. Subsequently, the patient underwent transurethral resection of bladder tumor (TURBT), and pathology demonstrated an invasive urothelial carcinoma with both conventional histology and variant plasmacytoid histology (Figure 1B). The plasmacytoid cells were poorly cohesive and had eccentric hyperchromatic nuclei with eosinophilic cytoplasm and occasional signet ring-like cells with intracytoplasmic mucin. The patient underwent chemotherapy with four cycles of MVAC (methotrexate, vinblastine, doxorubicin, cisplatin) with surgery (radical cystectomy with removal of ovaries/fallopian tubes/uterus/anterior 1/3 of the vagina/bilateral pelvic lymph nodes + ileal conduit creation). Pathology revealed residual invasive urothelial cell carcinoma with plasmacytoid histology, invasive into the superficial detrusor muscle. Six months later, the patient presented to the ED as stated above.

**Figure 2:**
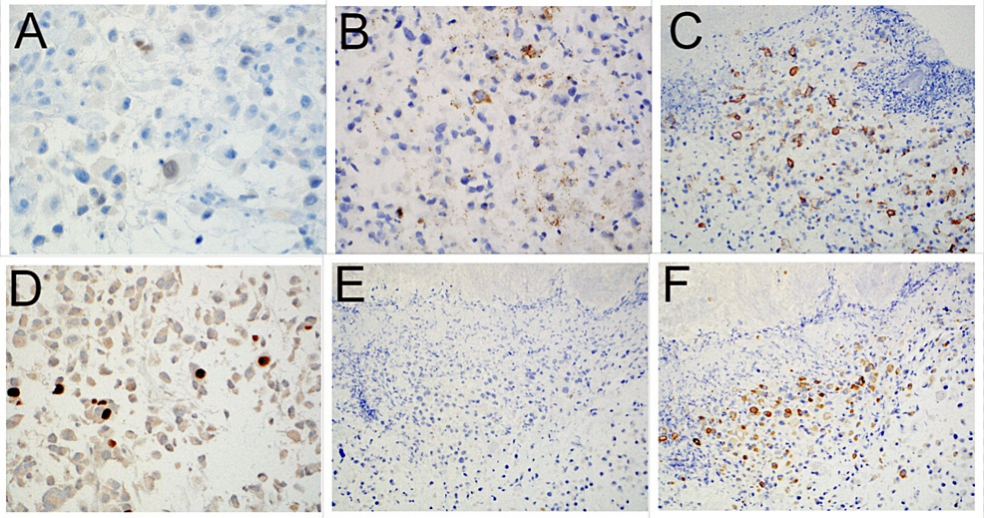
Immunohistochemical phenotype of the carcinoma in the lamina propria of the duodenum: (A) GATA3 (60x), (B) CK903 (20x), (C) CD138 (20x), (D) p63 (40x), (E) CK20 (20x), and (F) CK7 (20x). GATA3: GATA binding protein 3; CK: cytokeratin; CD: cluster of differentiation; p63: tumor protein 63.

**Figure 3:**
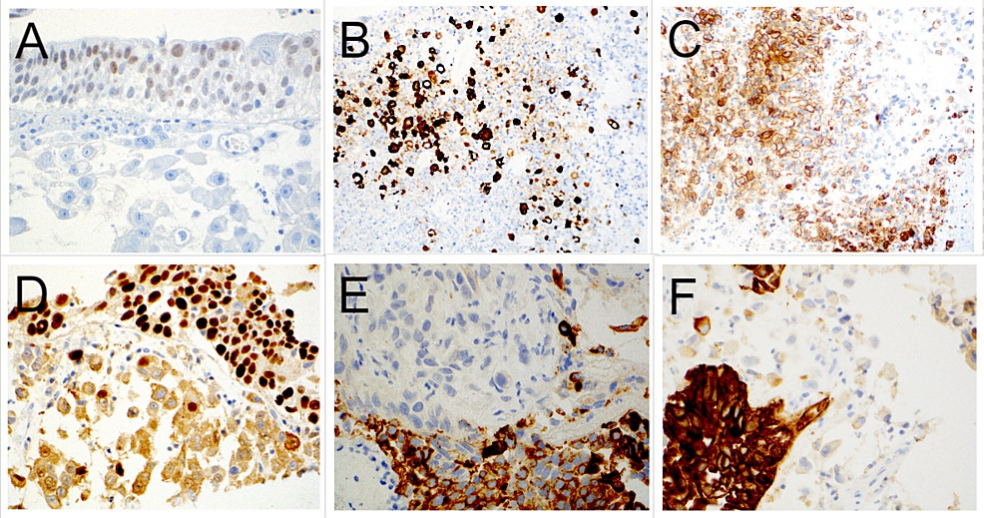
Immunohistochemical phenotype of the plasmacytoid component of the urothelial carcinoma present in the TURP specimen: (A) GATA3 (40x), (B) CK903 (20x), (C) CD138 (20x), (D) p63 (40x), (E) CK20 (40x), and (F) CK7 (40x). TURP: transurethral resection of the prostate; GATA3: GATA binding protein 3; CK: cytokeratin; CD: cluster of differentiation; p63: tumor protein 63.

The duodenal biopsy histopathology was compared to the prior TURBT and cystectomy pathology specimens, revealing an identical histomorphology of plasmacytoid and signet ring-like cells (Figure 1A, B). Knowing this previous history, additional immunohistochemical stains were performed on both the duodenal and TURBT biopsy specimens (Table 1). Interestingly, the duodenal biopsy was negative for GATA-3 and CK20 but positive for CK7, CK903, cluster of differentiation (CD) 138, and partially for tumor protein 63 (p63) (Figure 2A-F). Retrospective immunohistochemical analysis staining of the TURBT specimen demonstrated an identical immunoprofile in the plasmacytoid areas distinct from the conventional urothelial carcinoma component, which was GATA3 positive (Figure 3A-F). Ultimately, through comparison to the previous urothelial carcinoma histomorphology and demonstration of similar immunoprofiles supportive of a urothelial origin, the duodenal biopsy was determined to be metastatic PUC and not a primary GI small bowel signet ring carcinoma. Additionally, a biopsy of the large adrenal gland mass was also metastatic PUC.

**Table 1:** Immunohistochemical phenotype of the plasmacytoid cells/signet ring-like cells within the duodenum and the bladder. While GATA-3 is negative in both, the same overall patchy CK7, CK903, CD138, and p63 patterns were seen within the tumor cells, compatible with urothelial origin. Interestingly, the in situ and conventional urothelial carcinoma components retained positivity for both GATA-3 and CK20. GATA3: GATA binding protein 3; CK: cytokeratin; CD: cluster of differentiation; p63: tumor protein 63.

	GATA-3	CK-7	CK-20	P-63	CK-903	CD-138
Duodenum	-	+	-	+	+	+
Bladder	-	+	-	+	+	+

The patient was noted to have worsening weakness and hypotension and continued to have worsening anemia with a hemoglobin level of 4.8 g/dL. The patient received a packed red blood cell transfusion, and an upper GI endoscopy showed a large malignant appearing duodenal ulcer with diffuse friability with oozing blood at a few sites treated with hemoclip placement. Due to continued clinical decline, after detailed interdisciplinary discussions, the patient underwent interventional radiology (IR)-guided coil embolization of the gastroduodenal artery. Even after the procedure, the patient continued to bleed with worsening anemia despite blood transfusions. Subsequently, palliative care was also consulted, and after detailed informed discussions, the patient and family chose comfort care only without any other aggressive or invasive interventions. The patient was transferred to the palliative care unit where the patient passed away after a few days. 

## Discussion

Urothelial carcinoma is common in developed countries and is primarily related to smoking and carcinogen exposure [1]. In the United States, it is the fourth most common cancer in males and is predicted to account for nearly 7% of all new cancer cases in 2020 [2]. The estimated rate of incidence in males is four times higher than that for females; however, females tend to present at a higher clinical stage, are more locally aggressive, and are more likely to have lymph node metastases on presentation [3-5]. This female patient initially presented with hematuria, dysuria, and urinary frequency and was eventually found to have a bladder mass. TURBT demonstrated a urothelial carcinoma with involvement of the detrusor muscle, and CT imaging eventually identified transmural extension with involvement of the anterior uterine myometrium (cT4a), findings consistent with a high clinical stage on presentation.

The World Health Organization (WHO) recognizes urothelial carcinoma as having multiple histologic variants, some with different clinical behaviors and responses to treatment modalities [6]. PUC is one such variant. This particular histological variation of urothelial carcinoma is thought to represent anywhere between 1% and 4.9% of all invasive urothelial carcinomas [7]. The histological appearance is that of dyscohesive single cells with eccentric and hyperchromatic nuclei, eosinophilic cytoplasm, and signet ring-like cells with occasional intracytoplasmic mucin. These are the same histomorphological findings of the plasmacytoid component in the TURBT specimen as well as the malignant cells found within the duodenal biopsy (Figure 1A, B). Also unique to this histological variant, PUC has a tendency for intraperitoneal spread, including serosal surfaces, owing to more aggressive behavior [8]. PUC has also been shown to have high recurrence rates with shorter overall survival when compared to conventional-type urothelial carcinoma [4,5]. Very rare reports have documented metastases to the duodenum [9]. This patient had received neoadjuvant chemotherapy, followed by cystectomy with en bloc hysterectomy and anterior vaginectomy. Six months after surgery, she presented with anemia and was found to have duodenal wall thickening and a large adrenal mass. An endoscopic biopsy of the duodenal mass confirmed metastasis of the PUC. The close interval recurrence and nontraditional metastatic patterns are not unusual for this histologic variant of urothelial carcinoma. However, the duodenal site of metastasis is very rare and, in isolation, is very hard to distinguish from a potential primary GI small bowel signet ring cell carcinoma on histological grounds alone and may necessitate the aid of immunohistochemical stains.

Immunohistochemical staining patterns for conventional urothelial carcinoma are well characterized. One such immunohistochemical stain is GATA-3. GATA-3 expression in primary and metastatic urothelial carcinomas, including the plasmacytoid variant, has been observed in up to 80-90% of cases [10-12]. Thus, immunostaining with GATA-3 in evaluating for potential metastatic PUC is of great utility. Uniquely, the GATA-3 immunohistochemical stain was negative in the duodenal biopsy, and for this reason, the duodenal carcinoma was initially considered primary to the duodenum. Retrospective staining of the original bladder tumor in the TURBT specimen demonstrated GATA-3 positivity within the conventional urothelial carcinoma and in situ components; however, the plasmacytoid component of the original bladder tumor was negative for GATA-3 (Figure 3A). This highlights the importance of clinicopathologic correlation and review of prior surgical pathology specimens, despite site-specific immunohistochemical stains with known high sensitivities and specificities, like GATA-3. 

Before GATA-3, panels of immunohistochemical stains were used to support urothelial origin and included combinations of CK7, CK20, and p63, among others. Paner et al. led early attempts at defining the immunohistochemical profiles of conventional urothelial carcinoma as well as specific histologic variants, including plasmacytoid [11]. In this study, 14 total plasmacytoid variants were stained with GATA-3, CK7, CK20, and p63, among others, and rates of positivity were 100%, 70%, 60%, and 50%, respectively. As mentioned above, this plasmacytoid urothelial component was negative for GATA-3, but the duodenal biopsy and the original TURBT were also stained with CK7, CK20, and p63; both were negative for CK20 and demonstrated patchy positivity for CK7 and p63, consistent with a urothelial origin (Table 1). Lastly, it has been shown that PUC is CD138-positive, a traditional immunostain to mark plasma cells. This could also lead to a misdiagnosis of a plasma cell neoplasm if a broad immunohistochemical panel is not employed [13,14].

This case report highlights several important principles and potential pitfalls that could result in misdiagnosis and inappropriate patient treatment. The first paradigm that must be stressed is the importance of clinical history. In isolation, the duodenal biopsy demonstrated dyscohesive cellular proliferation within the lamina propria with signet ring-like cells. The diagnosis of a primary GI tract signet ring cell carcinoma can at times be difficult on endoscopic biopsy. One must keep in mind the potential for metastases, especially in women, with the possibility of lobular carcinoma of the breast and, as demonstrated in this case, PUC. By reviewing the patient’s clinical history and previous pathology, pertinent differential immunohistochemical stains can be included. The second paradigm involves the limitations of immunohistochemical stains. The duodenal biopsy was stained with GATA-3 and was negative. While GATA-3 has high sensitivity and specificity in metastatic urothelial carcinoma, including PUC, it is not 100%. Concordant with the duodenal biopsy, GATA-3 was also negative in the plasmacytoid component of the primary bladder tumor biopsy but positive in the conventional and in situ components. Recognizing the limitations of immunohistochemical stains, and even comparing them with previous biopsies, can provide valuable differential information. 

Lastly, not forgetting about the more classic immunostain to help establish urothelial origin is of utmost importance. A panel of CK7, CK20, and p63 can be just as discriminating as GATA-3, akin to mammaglobin and BRST-2 in breast carcinoma (in lieu of GATA-3) or prostate-specific antigen (PSA) and prostate-specific alkaline phosphatase (PSAP) in prostate adenocarcinoma (instead of NKX 3.1). In this case, while GATA-3 was negative, there was CD138 positivity and patchy CK7, keratin 903, and p63 positivity. In addition to the negative CDX-2, the overall immunohistochemical profile was helpful in distinguishing this as a metastasis versus a small bowel primary signet ring cell carcinoma.

## Conclusions

In conclusion, this case highlights several important features of PUC, including the more aggressive clinical course and nontypical routes of metastatic spread. In a limited biopsy specimen such as a duodenal biopsy, discriminating between a small bowel primary signet ring cell carcinoma and a metastatic carcinoma required careful correlation with clinical history, comparison to the primary bladder tumor histomorphology, and incorporation of all the immunohistochemical tools to support a metastasis of urothelial origin. In isolation, endoscopic biopsy of the duodenal wall thickening is fraught with the possibility of misdiagnosis as primary signet ring cell carcinoma and the potential to lead to clinical mismanagement. This case helps expand the histologic differential diagnosis of signet ring cell carcinoma, specifically PUC, and the potential pitfalls of using specific immunohistochemical stains alone. 
